# Flocks in focus: Automated video analysis of spatial behavior for stress detection in aviary-housed laying hens

**DOI:** 10.1016/j.psj.2025.105933

**Published:** 2025-10-01

**Authors:** Lara Amber van Veen, Anna Cornelia Maria van den Oever, Elisabeth Anna Maria Graat, Tom Van Hertem, Niels Demaître, Bas Kemp, Henry van den Brand

**Affiliations:** a Vencomatic Group, Meerheide 200, 5521 DW Eersel, The Netherlands; b Adaptation Physiology Group, Wageningen University & Research, P.O. Box 338, 6700 AH Wageningen, the Netherlands; c Experimental Poultry Centre, Province of Antwerp, Poiel 77, 2440 Geel, Belgium

**Keywords:** Spatial behavior, Flock-level monitoring, Laying hen, Stress detection, Behavioral consistency

## Abstract

Understanding stress responses in laying hens is crucial for improving welfare in commercial systems, yet real-time behavioral indicators remain underexplored. This study evaluated the use of automated spatial behavior monitoring to detect stress-induced behavioral changes in commercial-density laying hen flocks housed in aviary systems. The objectives were to (1) visualize and quantify vertical movement patterns and litter use over time and across flocks, and (2) assess behavioral changes in response to 3 stress contexts: visual (predatory bird), auditory (thunder sound), and frustrative (delayed feeding). Four flocks of 38-week-old hens were exposed weekly to each stressor over a 10-week period. Video data were analyzed using Python-based algorithms to assess vertical movement and litter use 1 hour before and after stress exposure. The effects of different stressors on vertical movement were analyzed using a Generalized Linear Mixed Model (**GLMM**), treating the experimental groups as biological replicates and including week as a random effect to account for temporal variation. Vertical movements significantly decreased during predator exposure (−8 movements/min, P < 0.001), but increased during thunder exposure (+6 movements/min, P < 0.001), delayed feeding (+1 movement/min, P < 0.005), and during the first 5 minutes of delayed feed supply (+3 movements/min, P < 0.001). GLMM analysis showed that litter use declined across all stress conditions, with the lowest densities recorded during predator and thunder exposure. Consistency of temporal patterns in spatial behavior varied across days, behavioral measures (i.e., vertical movement and litter use), and within the observation period. Significant differences in within-period consistency were confirmed using Friedman tests (P < 0.001), suggesting a potential link with induced stress. Vertical movement was most consistent in the afternoon in the absence of external disturbances. Stress exposure increased vertical movement consistency during recovery, potentially indicating synchronized vigilance. In contrast, litter use was more variable and sensitive to environmental changes. While this makes litter use less reliable as a standalone stress indicator, it might be a useful health and welfare indicator during predictable periods of the day, such as post-laying morning hours. These findings suggest that vertical movement consistency may serve as a promising behavioral indicator of stress recovery, while litter use patterns could inform welfare assessments during stable daily periods.

## Introduction

Spatial behavior, which involves space-use and movement in their confined environments, offers valuable insights into the dynamics and welfare of laying hens. Behaviors, such as locomotion and range use, are commonly linked to positive welfare, with longer and more frequent outdoor stays associated with improved well-being (Gebhardt-Henrich et al., 2014). Spatial behaviors can also be linked to negative welfare. Disrupted spatial behavior, such as altered locomotor rhythms, may contribute to issues like feather pecking (Bessei et al., 2023; Rodenburg et al., 2017).

Because welfare is closely linked to stress, spatial behavior may also reflect stress responses. Stress, defined as disruptions to physiological or psychological homeostasis, can originate from predation threats, social stress, or abiotic factors, like noise (Morgan & Tromborg, 2007). For example, perceived predation threats can lead to gregarious nesting (Riber, 2012) and increased night perching (Olsson & Keeling, 2000), while social stress due to e.g. feed competition may alter location preferences and hen distribution (Sirovnik et al., 2021). Noise stimuli increased fearfulness of different laying hen breeds, even after a single exposure of 60 mins with a noise level of 90 dB (Campo et al., 2005), and led to avoidance behavior (Hamm, 1967). These examples suggest that spatial behavior is sensitive to environmental stressors and might be indicative of experienced stress.

Although spatial behavior measures show daily consistency in hens (Rufener et al., 2018), large individual differences exist, which might reflect personality differences (Montalcini et al., 2023b). Because welfare is experienced individually (Fraser, 2008), individual monitoring is valuable, but resource-intensive and challenging to implement on a larger scale. Tracking devices (Montalcini et al., 2023a) or manual observation are common methods (Ciarelli et al., 2023) to assess spatial behavior, but flock-level automated monitoring may offer a non-invasive, scalable alternative. Group-level movement patterns are shaped by resource availability and social dynamics. For instance, feeding synchrony and clustering often reflect resource competition (Collins et al., 2011), and fear responses can spread socially, indicating emotional contagion (de Haas et al., 2012; Edgar & Nicol, 2018). Such contagion may affect spatial behavior at the flock level during stress, inducing a shared behavioral fear response among hens in large groups.

While laying hen spatial behavior has been studied in experimental and low-density settings (Yang et al., 2023), its potential for stress detection in commercial settings remains underexplored. This study investigated whether or not automated spatial monitoring in aviary systems can detect stress-induced behavioral changes in commercial flocks. Specifically, it examined vertical movement between aviary tiers and litter use across time and in response to 3 acute stress contexts: visual, auditory, and frustration-based.

## Materials and methods

### Ethical statement

All experimental procedures were approved by the Ethical Committee of the Province of Antwerp (under authorization number EC PP 2024-1; Provincie Antwerpen, Belgium) and all corresponding ethical guidelines were followed.

### Animals and housing

In total, 3840 ISA Brown pullets were reared at a commercial farm and were transferred simultaneously at 18 weeks of age to the Experimental Poultry Centre (Geel, Belgium). Laying hens were distributed across 4 pens, which were located in 2 laying hen compartments separated by a central corridor. The flocks in the 4 pens are referred to as “experimental group” further on. Experimental groups 1 and 2 (Compartment 1) were physically separated by a double wire fencing, while visual, auditory, and olfactory cues between them were not restricted. The same conditions applied to experimental groups 3 and 4 (Compartment 2).

Each experimental group was equipped with a single row, 3-tiered aviary system (Bolegg Terrace, Vencomatic Group, the Netherlands), housing 960 hens with intact beaks. The floor was covered with wood shavings, and measured 9.2 m by 7.2 m per experimental group. This resulted in a stocking density of 8.9 hens/m^2^ of usable area, which is in line with commercial conditions (European Commission, 2023). The housing system ensured free movement of hens across and underneath the aviary system. The 3 aviary tiers were designed as follows: a manure belt and a feeding line (lowest tier); group nests and nipple drinkers (middle tier); perches, 2 feeding lines and a manure belt (upmost tier).

Climate, feed and water provision and the light schedule were controlled per compartment. Artificial light was provided from 3:00 to 18:00 h (15 hours of light). The maximum light intensity was maintained at 35 lux. Manure was collected from the manure belts twice a week (Tuesdays and Fridays). Water was provided ad libitum using nipple drinkers. A commercial diet was provided at 3:00, 6:30 and 10:00 h at the lowest tier and the 2 feeding lines at the upmost tier, at 13:00 and 16.05 h at the lowest tier, and at 14:30 h at the 2 locations on the upmost tier.

### Experimental treatments

The experimental period started when hens were 38 weeks of age and lasted 10 weeks. Each week, 3 stressors were applied in all 4 experimental groups on separate days.

***Visual stressor.*** On Mondays at 15.30 h, a fake predatory bird was flown over the litter on the left side of the pen. The predatory bird was presented as silhouette of a hawk, as in Zeltner & Hirt (2008), with dimensions of 0.95 cm x 0.45 cm. When not flying, the bird rested inside a wooden box at 3.60 m height to ensure invisibility for the hens. To start stress induction, one researcher entered the compartment. After a 10 min habituation period, the fake bird was released every time at the exact same time during the experimental period. The bird was released via a declining cable ending at 2.70 m height. The fake bird was released for a single flight (1.32 m/s) and rested above the litter at the end of the track in sight of the laying hens. Five mins after bird release, the predatory bird was gently pulled back within 1 minute, leading to a total stress duration of 6 min (15:30-15:36 h). The predatory bird then disappeared out of sight again in the wooden box.

***Frustrative stressor.*** On Wednesdays, the regular feeding round at 10:00 h was delayed by an hour until 11:00 h in all experimental groups. At 11:00 h, the regular amount of feed was provided to the hens.

***Auditory stressor.*** On Fridays at 15:30 h, the hens were exposed to a 30 second recording of hailstorm and thunder at 92dB (Putyora et al., 2023). Two speakers hung at 2.75 m height in the compartments and were connected to 1 amplifier. The recording was played from a laptop outside of the poultry house. From week 5 of the experiment onward, the sound fragment of hailstorm and thunder was reduced in intensity, resulting in sound of 88 dB for 30 seconds.

### Video recordings

**Fig. 1:**
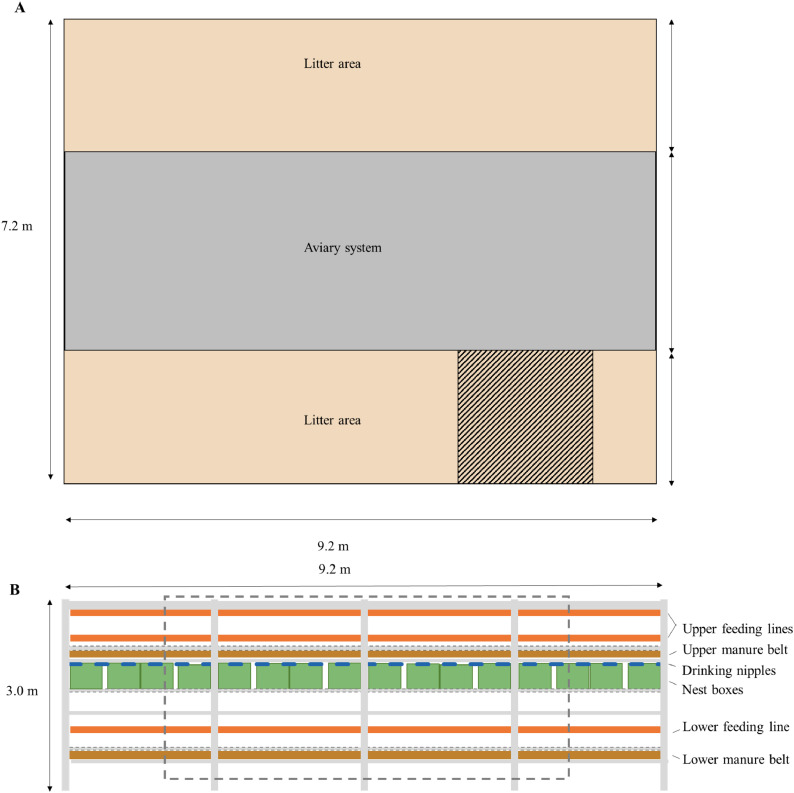
**Visual representation of camera recording views in the experimental groups.** Camera field of views are marked. A = camera 1, a top-view on the litter area (4 m^2^); B = camera 2, a side view of the aviary system (16.5 m^2^).

Three infrared cameras (Hikvision, DS-2CD2021G1-I, China) were placed at the left side of the aviary system per experimental group. Camera 1 recorded videos perpendicular on the litter area at a height of 2.1 m for analysis of litter use (Fig. 1A). Camera 2 recorded videos directly facing the side of the aviary system at a height of 1.95 meters for analysis of vertical laying hen movement (Fig. 1B). A third camera hung at 1 meter height on the entrance door of the compartments and viewed over the length of the litter. The videos from camera 3 were used only for behavioral observation of the hens and their environment aid flock observations for ethical control reasons and were not used in any analysis in this study.

Video recordings were continuously stored on a network video recorder (Hikvision, DS-7616NXI-K2, China) during the full study period. Video data was extracted for data collection 1 hour pre-stress induction, during stress and until 1 hour post-stress induction. This resulted in 2 hours of recording around visual and auditory stress and 3 hours of recording around feed frustration. From week 7 onwards, it was decided to extract video data from Tuesdays around regular feeding at 10:00 until 11:04 to allow comparison of hen behavior during frustrated feeding (Wednesday) and regular feeding (Tuesday). In total, 73 hours of video data were of interest per camera, and thus 584 h for behavioral analysis (camera 1 and 2).

### Behavioral analysis

**Fig. 2:**
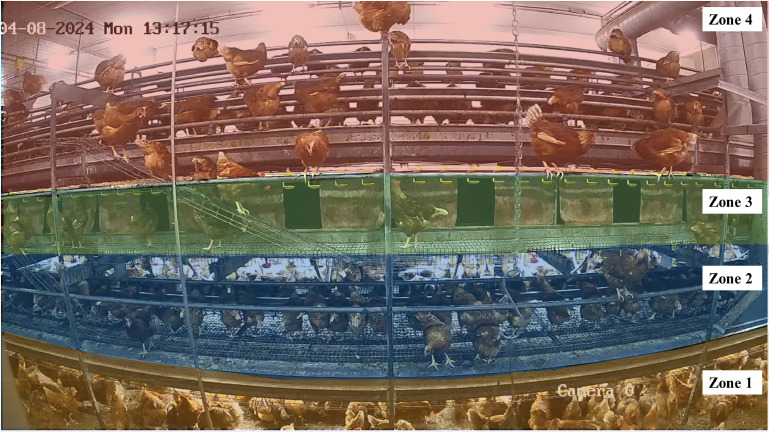
**Overview of the 4 horizontal zones for vertical movement analysis.** The computer vision analysis tool counted vertical movements between horizontal zones.

***Vertical movement.*** A computer vision analysis tool was developed to count the number and direction of vertical movements between 4 horizontal zones (3 tiers and the litter area) in the aviary system (Fig. 2; based on Camera 2 observations). The tool was built in Python (v3.9.13), with modules OpenCV, NumPy and Matplotlib. Videos were processed using FFmpeg 5.1.1 video processing software, and horizontal zones were annotated with Labelme for each individual video. Each frame (4 frames/s) was converted into greyscale and blurred to smoothen the image and remove noise. The software assessed vertical movements between zones based on computing pixel differences between consecutive frames, using the OpenCV’s absdiff method. Counted movements of objects (i.e. hens) between zones were saved into a .csv file, while the processed video frame with all annotated movements between zones were saved into .mp4. Horizontal zones were drawn in such a way that hens had to jump between tiers and the litter to let it count as movement. The hens walking on the stairs between zone 3 and 4 were not recorded.

***Henlitter use.*** A second computer vision analysis tool was developed in Python (v3.9.13) to detect and quantify laying hens across 2 regions of the litter in real-time (based on Camera 1 observations). YOLOv9-C (Wang et al., 2024) was used as object detection model. The model was initially trained on a comprehensive dataset of videos captured within the study farm, ensuring its reliability in recognizing and tracking hens under various conditions.

**Fig. 3:**
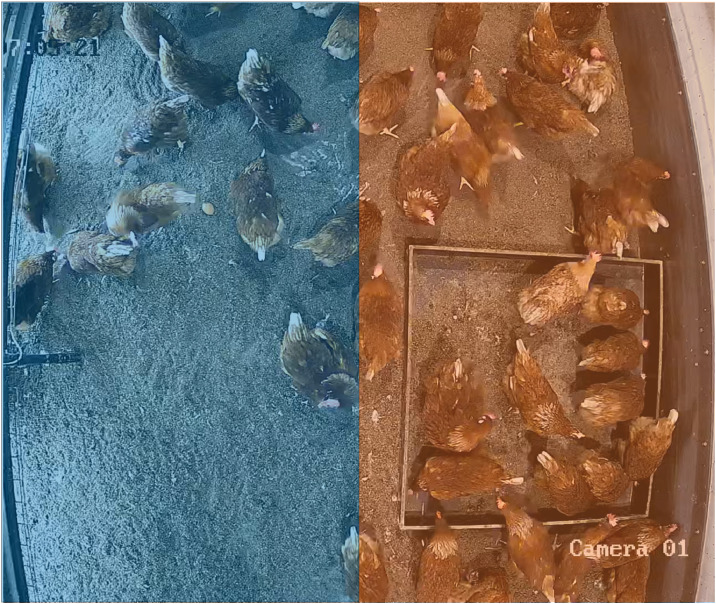
**Overview of the 2 litter zones for litter use analysis.** The computer vision analysis tool counted the number of hens per litter zone. Blue = system-side; Orange = wall-side.

Raw video recordings were processed, using FFmpeg 5.1.1. The videos were cropped to isolate the litter area, excluding visible walls and parts of the aviary system. Grids within the video frame were defined manually, with 2 zones (Fig. 3). This configuration facilitated detailed analysis of spatial distribution across different zones, i.e. close to and underneath the system, and close to the wall and the enrichment. After training, the YOLOv9-C model was converted to OpenVINO 2024.0.0 format to optimize real-time inference and thus enhance computational efficiency. The detection process involved generating bounding boxes around each detected hen, which enabled precise spatial distribution analysis across the defined zones. The system’s AI components produced 3 primary types of outputs: annotated videos displaying detection results, comprehensive Excel spreadsheets detailing hen counts and distributions across defined zones, and visual heatmaps illustrating hen movement and density over time.

### Software evaluation

The software for vertical movement analysis and litter use analysis were evaluated by manual observation of annotated videos. For each week, 1 video was randomly selected for evaluation across all experimental groups and experimental treatments. This resulted in 10 video evaluations regarding vertical movement analysis and 10 videos regarding litter use analysis. The 20 videos represented moments with varying levels of movements and animal densities in the system and on the litter between 9:00 – 12:00 h and 14:30 – 16:30 h. Per video, 3 mins were selected for manual observation. Evaluation was performed with a confusion matrix. Precision, recall and the F1-score were calculated according to the following equations (Paneru et al., 2024):

(1) Precision=TP/(TP+FP)

(2) Recall=TP/(TP+FN)

(3) F1−score=(2xPrecisionxRecall)/(Precision+Recall)

where TP is the number of true positives, meaning that the software correctly detected a vertical movement between tiers or where the software correctly detected the number of hens per zone. FP is the number of false positives, where the software incorrectly detected vertical movement between tiers or the software detected too many hens per zone. FN is the number of false negatives, where a vertical movement between tier was not recognized or where too few hens were recognized per zone. The F1- score represents the harmonic mean of precision and recall, providing a balanced measure of the software’s performances.

### Performance and welfare measurements

Eggs were graded and sorted daily by the Staalkat Alpha 70 egg grader (Sanovo Technology Group, Denmark), except on Sunday. Hen performance was monitored per experimental group, with daily figures of laying percentage, average egg weight, average water consumption, average feed consumption and mortality. Further, in all experimental groups, the body weight of hens was monitored continuously by automated weighing scales. Performance numbers from the experimental groups were compared with the norm for ISA Brown hens held in aviary systems.

In all experimental groups, the poultry red mite infestation level was monitored once per week, using 4 8cmx8cm cardboard traps per experimental group divided over several locations in the poultry house. During the full duration of the study, no poultry red mites were detected. During daily control of the experimental groups, weak or sick hens were removed from the flock. As part of the ethical framework of the study, comprehensive welfare assessments were performed. Welfare was assessed weekly per experimental group using a protocol for the early onset detection of injurious pecking (Van den Broeck et al., 2022). The feather score was additionally scored monthly according to the Tauson method (Tauson et al., 2009), together with scoring keel bone deformations and comb wounds.

### Statistical analysis

***Temporal dynamics of spatial behavior.*** All statistical analyses were performed within R-4.2.2. (R Core Team, 2021). The first step was to visualize data of vertical movement and litter use in time. Data were averaged per min across weeks, groups, and days, generating an average (±SD) movement curve for the 3 observation days, with standard deviations across 10 weeks and 4 experimental groups. Activity categories were defined to allow detailed time pattern analysis. Categories related to feeding included:

• 2-min feed belt operation plus 3-min initial feeding ((Delayed) Feeding 1 and Feeding 2).
• 5-min feeding sounds from neighboring non-experimental compartments, which were audible in the experimental groups (Feeding sound).

Stress-related categories were structured around specific experimental stressors:

• Monday: Predator cue 1 (3-min initial startling response) and Predator cue 2 (3-min response to prolonged exposure).
• Wednesday: Four 15-min Frustration stages (0–15, 16–30, 31–45, 46–60 mins after the start of delayed feeding) to differentiate between phases of frustration.
• Friday: 1-min Thunder sound response.

Resting periods were defined for all remaining minutes, with 2 periods before and 2 periods after stress events to balance activity durations. During these periods, no management or experimental interventions occurred, allowing hens to engage in active behaviors (e.g., dustbathing, relocation) or inactive behaviors (e.g., standing on litter or in the aviary).

***Weekly consistency in spatial behavior.*** To evaluate weekly consistency in spatial behavior, descriptive statistics of the vertical movement and the litter use were calculated for each activity category across the different weeks after averaging for experimental group. This included the median with the interquartile range (**IQR**), **Q1** (first quartile), **Q3** (third quartile), and quartile coefficient of dispersion (**QCD**) between weeks, and the weekly minimum, and weekly maximum vertical movement and litter use respectively. A Friedman’s test (Friedman, 1937) from the R package ‘rstatix’ (Kassambara, 2021) was used to compare vertical movement and litter use between the defined activity categories per day, averaged across experimental groups and with week as blocking factor to control for week-specific effects on spatial behavior measures. In the case of a significant effect, pairwise post hoc comparisons between the different activity categories were performed, using the exact all-pairs comparisons test with False Discovery Rate control to adjust P-values.

***Stress-induced alterations in spatial behavior.*** To evaluate the relationship between vertical movement and litter use in the stress contexts, generalized linear mixed models (**GLMM**) from the R package ‘glmmTMB’ (Brooks et al., 2017) were used. Outcome variables were the total number of vertical movements and hen numbers on the litter per min counted separately per experimental group and week, and tested per individual stressor. Data were analyzed using a negative binomial regression to account for the high variance observed in vertical movement and hen density counts relative to their means. Week was included as a random effect to address the repeated measurements over time. Experimental groups were handled as biological replicates. Stress phases (pre-, during and post- stress) were included as fixed effects.

The model was used across different durations pre-stress and post-stress, to study respectively short-term and long-term effects of stress on spatial behavior measures. Short-term responses to visual and auditory stress were analyzed by looking at the 5 minutes before stress induction (pre-stress), the entire duration of stress induction (during stress), and the first 5 minutes after stress induction (post-stress). Long-term responses to these stressors considered a longer timeframe, including the 25 minutes before stress, the full stress period, and the first 25 minutes after stress.

For delayed feeding, short-term responses were measured during the 5 minutes before stress, either the first or last 5 minutes of the stress period, and the first 5 minutes after stress. Long-term responses covered 30 minutes before stress, either the first or last 30 minutes during stress, and the first 30 minutes post-stress.

Mean performance traits and mean deviation from the norm (feed intake, laying percentage, egg weight, hen body weight and cumulative mortality) in the experimental groups were calculated for 3 periods of 5 weeks, which were the pre-experimental period (wk. 33 to 37 of age), experimental period 1 (wk. 38 to 42 of age) and experimental period 2 (wk. 43 to 47 of age).

## Results

### Software evaluation

Software evaluation showed a recall of 0.84 for detecting vertical movements across groups and stress contexts, with an F1-score of 0.89 and precision of 0.95. For litter use, recall was 0.90 across groups and stress contexts, accompanied by an F1-score of 0.94 and precision of 0.99.

### Temporal dynamics of spatial behavior

**Fig. 4:**
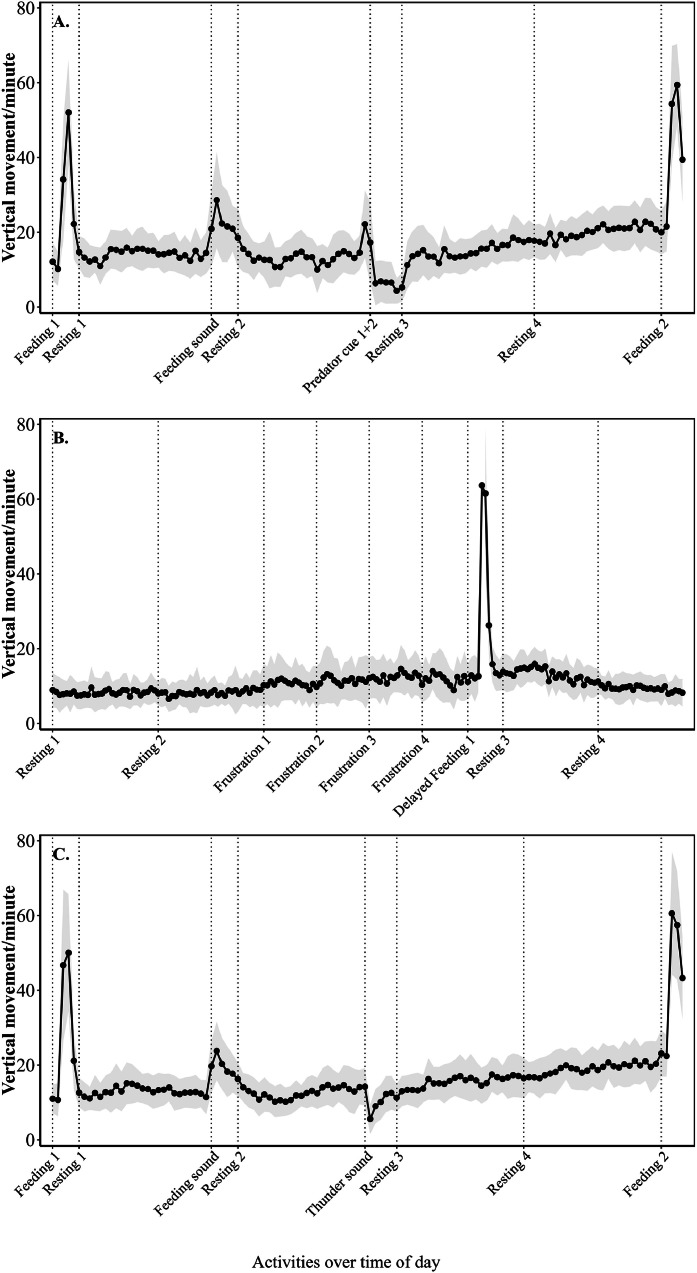
**Average vertical movement pattern per min across 4 experimental groups of laying hens during 10 consecutive weeks (38-47 weeks of age) across activities.** Dotted lines indicate the start of an activity. A = Monday, in which a visual stressor was applied at 15:30 h; B = Wednesday, at which feed provision was delayed from 10:00 to 11:00 h; C = Friday, at which an auditory stressor was applied at 15:30 h. Standard deviation across groups and weeks is depicted in grey.

***Vertical movement.*** First, the average vertical movement was calculated for each week, group and day of the week (Supplemental Table 1). Then, vertical movement data were aggregated by calculating the average across experimental groups and weeks. Average daily vertical movement ranged from 9 to 21 movements/min. Average vertical movement of the 4 experimental groups demonstrated visible time patterns across the 10 observational weeks (Fig. 4). A reduction in vertical movements was seen during predator exposure (Fig. 4A) and during thunder sound exposure (Fig. 4C), while vertical movements increased during delayed feeding (Fig. 4B)

**Fig. 5:**
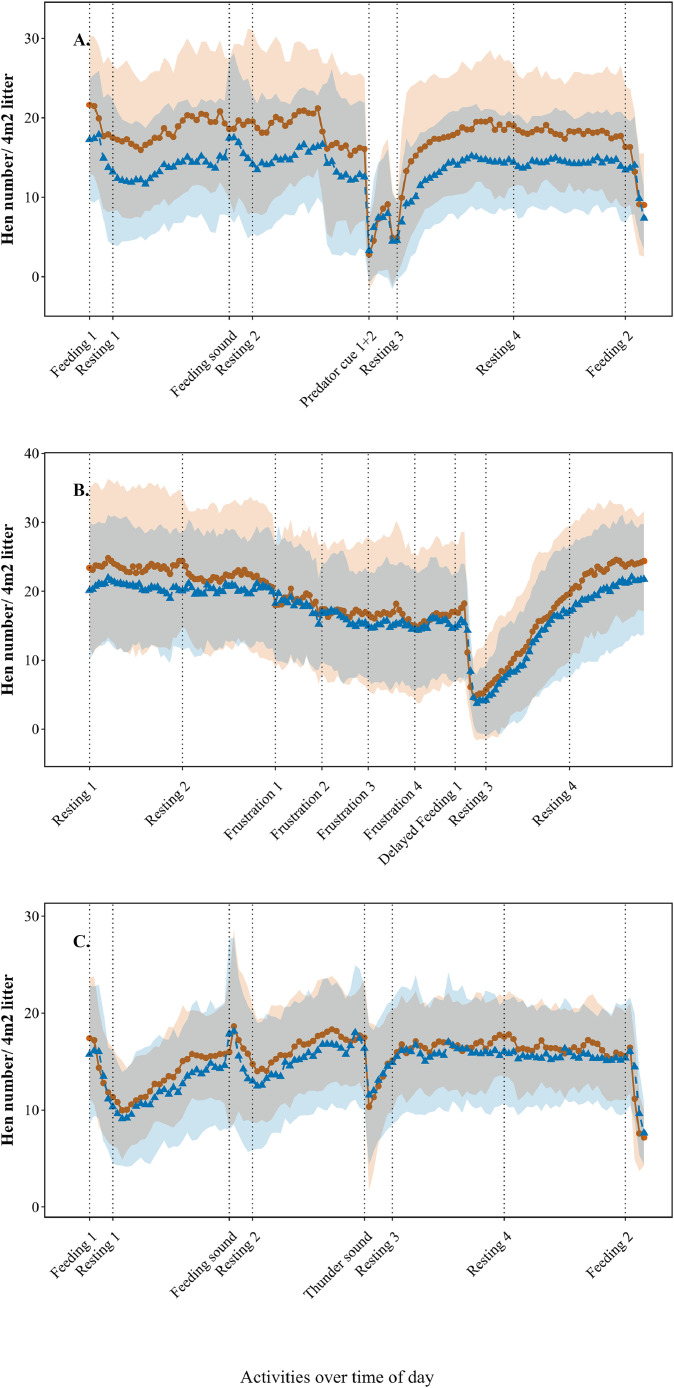
**Average hen number on 4m^2^ litter across 4 experimental groups of laying hens during 10 consecutive weeks (38-47 weeks of age) along the wall (●) and along the aviary system (▲) across activities**. Standard deviation across groups and weeks is depicted in orange and blue shading (with grey overlap). Dotted lines indicate the start of an activity. A = Monday, in which a visual stressor (predator bird) was applied at 15:30 h; B = Wednesday, at which feed provision was delayed from 10:00 to 11:00 h; C = Friday, at which an auditory stressor (thunder sound) was applied at 15:30 h.

***Litter use.*** Similarly, the litter use was calculated for each week, experimental group and day of the week (Supplemental Table 2), and then averaged across experimental groups and weeks. Average daily litter use ranged between 4 hens/m^2^ and 18 hens/m^2^. Fig. 5 shows the pattern of hen numbers on the litter in the 4 experimental groups, averaged across 10 weeks on 3 days, both along the aviary system and along the wall of the poultry house. A reduction in hen number on the litter was seen during predator exposure (Fig. 5A), during delayed feeding (Fig. 5B) and during thunder sound exposure (Fig. 5C).

**Fig. 6:**
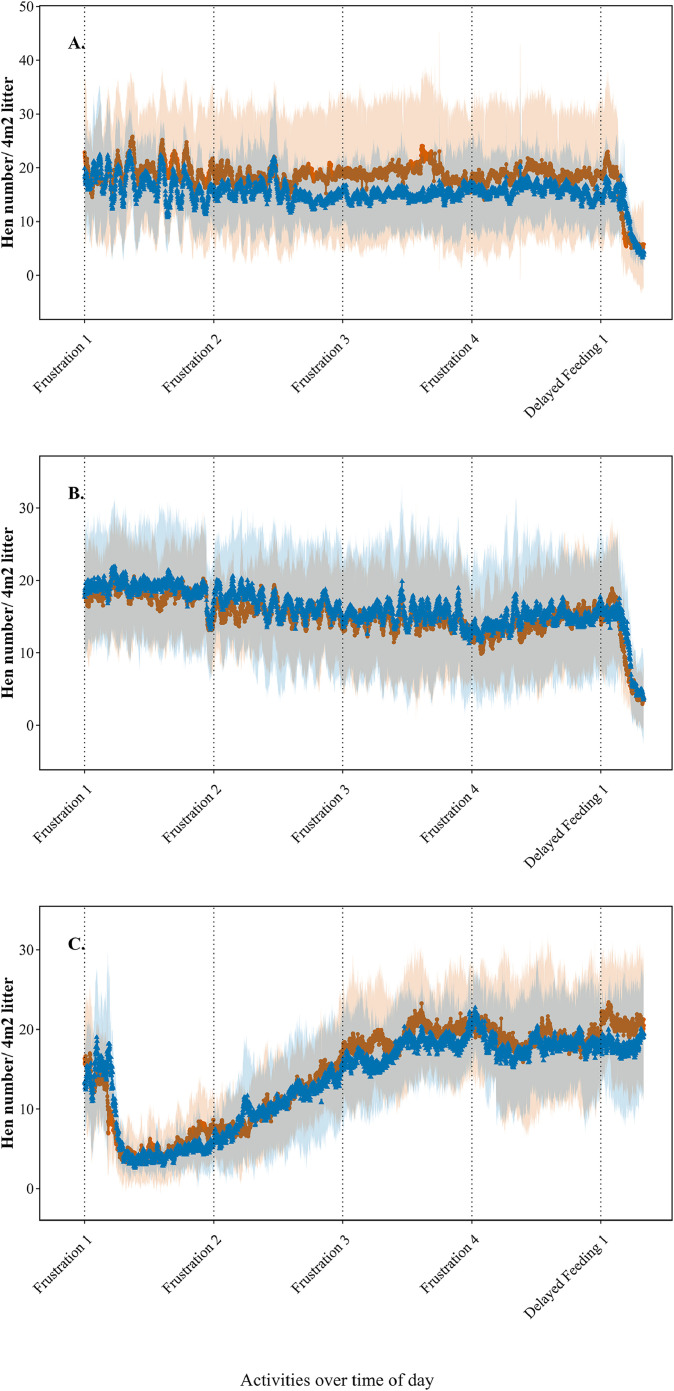
**Average litter use pattern per second across 4 experimental groups of laying hens (38-47 weeks of age) during 10 consecutive weeks on the litter along the wall (●) and along the aviary system (▲) between 10:00 and 11:04 h.** Standard deviation across groups and weeks is depicted in orange and blue shading (with grey overlap). A = Wednesday compartment 1, at which feed provision was delayed from 10:00 to 11:00 h. B = Wednesday compartment 2, at which feed provision was delayed from 10:00 to 11:00 h. C = Tuesday compartment 1 and 2, at which feed provision was not delayed as control.

On Monday, litter use differed in mean and standard deviation along the wall and aviary, with higher hen numbers on the litter area near the wall. However, litter use along the system and wall followed a similar time pattern on Monday, Wednesday, and Friday. On Wednesday, time patterns of litter use were different between experimental group 1 and 2 (Compartment 1) versus experimental group 3 and 4 (Compartment 2), specifically from 10:00 to 11:04 (thus during the delayed feeding period). An additional figure was made to illustrate the number of hens on the litter, showing variations per second along the wall and the system on Wednesday during delayed feeding in Compartment 1 (Fig. 6A) and Compartment 2 (Fig. 6B). For comparison, Fig. 6C presents data from both compartments on Tuesday when feeding was not delayed.

### Weekly consistency in spatial behavior

***Vertical movement.*** For each experimental group, the median vertical movement per minute was calculated per activity per week, along with the interquartile range (**IQR**) and the quartile coefficient of dispersion (**QCD**) to quantify variability between weeks. These values were then aggregated across groups to obtain overall medians and variability estimates per activity (Table 1). The weekly minimum and maximum vertical movements per minute and litter use per activity category are also displayed. On days with the predator cue and thunder sound cue, the QCD was highest during the stress-related activity categories (31.9% – 39.0%), with the lowest median vertical movement (5.0 – 9.3 movements/min). The weekly QCD was low (2.3% – 4.4%) during all Resting activities after exposure to visual and auditory stress. Median vertical movement/min was highest during Feeding 2 on the day of predator release (39.9 movements/min) and on the day of thunder sound exposure (41.3 movements/min). On the day of delayed feeding, the QCD was lower during Delayed Feeding 1 (3.3%) than during Resting, and the QCD of Resting after stress was lower than the QCD before stress. The QCD was highest during Frustration 2 (15.0%) and lowest during Frustration 1 (2.7%).

**Table 1:** **Descriptive statistics of vertical movement, averaged over 4 experimental groups of laying hens during 10 consecutive weeks (38 - 47 weeks of age).** Median including interquartile range (IQR), Q1 (first quartile), Q3 (third quartile), and quartile coefficient of dispersion (QCD) between Weeks, with Weekly Minimum, and Weekly Maximum vertical movement per min per activity category.

**Activity category**	**Duration (min)**	**Median vertical movement/min**	**IQR**^1^	**Q1-Q3**^2^	**QCD (%)**^3^	**Weekly minimum -maximum vertical movement/min**
**Visual stressor**						
Feeding 1	5	25.2	0.7	24.6 - 25.3	1.3	22.9 - 35.5
Resting 1	25	14.0	1.8	13.0 - 14.0	6.5	12.0 - 17.5
Feeding sound	5	23.9	6.3	18.4 - 24.7	14.6	17.5 - 30.5
Resting 2	25	13.1	1.7	12.5 - 14.2	6.4	12.0 - 17.3
Predator cue 1	3	9.3	6.1	6.5 - 12.6	31.9	5.8 - 16.5
Predator cue 2	3	5.0	2.2	3.7 - 5.8	22.8	2.6 - 11.3
Resting 3	25	14.0	0.6	13.6 -14.2	2.3	12.9 - 18.5
Resting 4	24	19.4	1.8	19.1 - 20.8	4.4	18.0 - 23.2
Feeding 2	5	39.9	5.3	36.9 - 42.1	6.6	31.6 - 42.7
**Delayed feeding**						
Resting 1	30	7.9	1.4	7.8 - 9.1	8.0	7.2 - 11.6
Resting 2	30	8.2	1.4	7.6 - 9.0	8.5	6.4 - 13.7
Frustration 1	15	10.8	0.6	10.6 - 11.2	2.7	9.1 - 15.4
Frustration 2	15	11.3	3.3	9.3 - 12.6	15.0	8.6 - 17.6
Frustration 3	15	11.9	1.8	10.9 - 12.7	7.8	10.3 - 17.1
Frustration 4	15	11.8	2.8	9.2 - 12.1	13.3	7.1 - 15.6
Feeding 1	5	34.6	2.3	33.9 - 36.2	3.3	32.4 - 38.2
Resting 3	30	13.3	1.4	12.6 - 13.9	5.2	11.7 - 15.5
Resting 4	30	9.0	1.1	8.6 - 9.7	5.9	7.7 - 10.9
**Auditory stressor**						
Feeding 1	5	27.1	3.7	26.0 - 29.8	6.7	22.8 - 33.6
Resting 1	25	12.7	1.8	12.2 - 14.0	6.7	11.3 - 14.2
Feeding sound	5	20.5	2.9	18.5 - 21.4	7.2	14.6 - 23.2
Resting 2	25	12.9	1.1	12.3 - 13.4	4.1	11.3 - 13.9
Thunder sound	1	8.1	5.4	4.3 - 9.7	39.0	2.5 - 13.8
Resting 3	27	15.0	1.0	14.6 - 15.6	3.2	13.1 - 16.8
Resting 4	26	19.2	0.9	18.6 - 19.5	2.3	17.7 - 20.3
Feeding 2	5	41.3	3.0	40.5 - 43.5	3.6	34.1 - 45.5

1 IQR = Interquartile range

2 Q1 = First quartile; Q3 = Third quartile

3 QCD = Quartile coefficient of dispersion

The Friedman test showed differences in vertical movement between activity categories on the day of visual stress (χ²(8) = 68.24, *P* = 1.10 × 10⁻¹¹, W = 0.95), frustrative stress (χ²(8) = 44.80, *P* = 4.02 × 10^-7^, W = 0.62), and auditory stress (χ²(7) = 64.93, *P* = 1.55 × 10^-11^, W = 0.93).

***Litter use.*** Similarly, the aggregated values of the median litter use were calculated for each activity, along with the IQR, the QCD, and the weekly minimum and maximum litter use (Table 2). On the day of predator exposure, the QCD of weekly litter use at Resting numerically increased after Feeding sound (25.9% to 31.3%) and decreased again after Predator cue 1 and 2 (29.5% – 22.2%). On the day of thunder sound exposure, Resting QCD decreased from 10.5% before auditory stress to 5.2% after auditory stress. On both the day of predator exposure and thunder sound exposure, the number of hens on the litter was lowest during stress (2-3 hens/m^2^ and 6 hens/m^2^ resp.), with the highest QCD during these moments. In general, the QCD was higher on the day of predator exposure than on the day of thunder sound exposure. QCD was higher during Feeding Sound than Feeding 1 and Feeding 2 on both the day of predator exposure and thunder sound exposure. On the day of delayed feeding, QCD was highest during Frustration 3 (27.5 %), followed by Frustration 4 (25.5 %). The Resting QCD was higher after frustration than before frustration. The highest maximum litter use was during Resting 1 on Wednesday morning, with 10 hens/m^2^. Pre-stress Resting QCD was lower on Wednesday (7.2% - 8.6%) than Monday (25.9 %– 31.3 %) and Friday (12.9% – 10.5%).

**Table 2:** **Descriptive statistics of litter use, averaged over 4 experimental groups of laying hens during 10 consecutive weeks (38-47 weeks of age).** Median including the interquartile range (IQR), Q1 (first quartile), Q3 (third quartile), and quartile coefficient of dispersion (QCD) between Weeks, with Weekly Minimum, and Weekly Maximum litter use per activity category.

**Activity category**	**Duration (min)**	**Median litter use/4m^2^**	**IQR**^1^	**Q1-Q3**^2^	**QCD (%)**^3^	**Weekly minimum -maximum litter use/4m**^**2**^
**Visual stressor**						
Feeding 1	5	30.3	14.9	28.0 - 42.9	21.0	24.1 - 57.0
Resting 1	25	28.2	16.6	23.6 - 40.2	25.9	20.6 - 48.6
Feeding sound	5	32.8	20.3	25.2 - 45.5	28.7	23.6 - 49.8
Resting 2	25	26.5	21.2	23.3 - 44.6	31.3	21.9 - 49.8
Predator cue 1	3	8.6	5.0	6.6 - 11.7	27.5	4.3 - 21.7
Predator cue 2	3	10.7	10.0	7.9 - 17.9	38.6	4.6 - 29.4
Resting 3	25	23.0	18.7	22.4 - 41.1	29.5	20.4 - 44.7
Resting 4	24	28.3	15.0	26.4 - 41.4	22.2	23.5 - 48.2
Feeding 2	5	20.1	11.8	19.1 - 30.0	23.6	16.3 - 38.7
**Delayed feeding**						
Resting 1	30	39.5	5.7	36.6 - 42.3	7.2	32.6 - 80.5
Resting 2	30	38.8	6.5	34.5 - 41.0	8.6	31.0 - 74.3
Frustration 1	15	33.1	9.8	29.9 - 39.7	14.1	27.9 - 60.7
Frustration 2	15	31.0	10.4	26.1 - 36.5	16.6	18.3 - 53.2
Frustration 3	15	29.3	16.0	21.1 - 37.1	27.5	15.6 - 61.7
Frustration 4	15	4.7	15.8	23.1 - 39.0	25.5	18.9 - 58.9
Feeding 1	5	19.4	9.6	18.4 - 28.0	20.7	17.0 - 40.4
Resting 3	30	15.6	9.0	14.1 - 23.1	24.2	13.7 - 45.3
Resting 4	30	37.1	10.6	36.1 - 46.8	12.8	33.3 - 60.9
**Auditory stressor**						
Feeding 1	5	29.4	6.6	26.2 - 32.9	11.2	21.2 - 37.1
Resting 1	25	25.0	6.7	22.5 - 29.2	12.9	18.8 - 31.6
Feeding sound	5	33.7	7.4	28.7 - 36.1	11.4	26.7 - 38.6
Resting 2	25	31.6	6.7	28.4 - 35.1	10.5	25.8 - 37.9
Thunder sound	1	24.6	17.2	13.9 - 31.0	38.2	7.5 - 36.8
Resting 3	27	31.5	3.4	30.6 - 34.0	5.2	26.7 - 39.0
Resting 4	26	31.7	6.2	29.9 - 36.1	9.5	26.2 - 38.1
Feeding 2	5	24.6	4.3	21.8 - 26.1	8.9	19.6 - 31.1

1 IQR = Interquartile range

2 Q1 = First quartile; Q3 = Third quartile

3 QCD = Quartile coefficient of dispersion

The Friedman test showed differences in hen number on the litter between activity categories on the day of visual stress (χ²(8) = 62.07, P = 1.823e-10, W = 0.86), frustrative stress (χ²(8) = 52.21, P = 1.535e-08, W = 0.72), and auditory stress (χ²(7) = 37.1, P = 4.49e-06, W = 0.53).

### Stress-induced alterations in spatial behavior

***Vertical movement.*** The exposure to the predatory bird induced a decrease (P < 0.001) in vertical movement from 15.6 movements/min before predatory bird exposure to 7.5 movements/min during predatory bird exposure (Table 3). Movement remained lower at 11.4 movements/min (P < 0.001) during the 5 mins after stress induction. On the long-term, predatory bird exposure resulted in a small, but significant increase in movement (P = 0.004) compared to the period before predatory bird-induced stress (Supplemental Table 3).

**Table 3:** Short term effect of visual (predator bird) and auditory (thunder sound) stressors on vertical movement of 4 experimental groups of laying hens during 10 consecutive weeks (age 38-47 weeks).

**Stressor**	**Duration**	**Movement/min**	**SE**	P*	**Week-effect**
**Visual stress**					
Before predatory bird	5 min	15.6	0.09		0.05 ± 0.23
During predatory bird	6 min	7.5	0.07	< 0.001	
After predatory bird	5 min	11.4	0.07	< 0.001	
**Auditory stress**					
Before thunder sound	5 min	7.3	0.06		0.01 ± 0.12
During thunder sound	1 min	12.6	0.05	< 0.001	
After thunder sound	5 min	14.1	0.06	< 0.001	

⁎ *P* <0.05 is significant difference compared to before predatory bird/thunder sound

The thunder sound increased (P <0.001) vertical movement from 7.3 vertical movement/min before stress to 12.6 vertical movements/min during stress, and vertical movement/per remained elevated (P < 0.001) during the 5 mins post stress (Table 3). On the long term, vertical movement remained elevated after thunder sound exposure (P < 0.001) with 5.5 vertical movements/min more after stress compared to before stress (Supplemental Table 3).

Vertical movements per min were increased from 9.0 vertical movement/min before delayed feeding to around 10 vertical movements per min during delayed feeding, both during the first 5 mins of delayed feeding (P = 0.005), and during the last 5 mins of delayed feeding (P < 0.001) (Table 4). The same patterns were seen for the long-term response (Supplemental Table 4).

**Table 4:** **Short-term effect of frustrative stress (delayed feeding) on vertical movement of 4 experimental groups of laying hens during 10 consecutive weeks (age 38-47 weeks).** Results are given for early frustration (the first 5 mins of delayed feeding) and late frustration (the last 5 mins of delayed feeding).

**Model factors**	**Duration**	**Movement/min**	**SE**	P*	**Week-effect**
*Early frustration*					
Before delayed feeding	5 min	9.0	0.05		0.002 ± 0.05
During delayed feeding	First 5 min	10.2	0.07	0.005	
After delayed feeding	5 min	12.9	0.07	< 0.001	
*Late frustration*					
Before delayed feeding	5 min	9.0	0.05		0.00 ± 0.0001
During delayed feeding	Last 5 min	10.3	0.07	< 0.001	
After delayed feeding	5 min	12.9	0.07	< 0.001	

⁎ *P* <0.05 is significant difference compared to before delayed feeding

Overall, the random intercepts for weeks were lower in the long-term model compared to the short-term model of vertical movement, together with relatively high standard errors in all models. This suggests that variation between weeks had a greater impact on the analysis of the short-term stress response, with significant uncertainty regarding the week effect across all analyses.

***Litter use.*** The exposure to the predatory bird induced a decrease (P < 0.001) in the hen number on 4m^2^ litter from 27.6 hens before predator exposure to 13.0 hens during predator exposure (Table 5). Hen number remained decreased (P < 0.001) during the 5 mins after stress induction with 17.7 hens on 4m^2^ litter. The same patterns were seen for the long-term response (Supplemental Table 5).

**Table 5:** Short term effect of visual (predator bird) and auditory (thunder sound) stressors on litter use of 4 experimental groups of laying hens during 10 consecutive weeks (age 38-47 weeks).

**Model factors**	**Duration**	**Hens/ 4 m^2^ litter**	**SE**	P*	**Week-effect**
**Visual stress**					
Before predatory bird	5 min	27.6	0.15		0.18 ± 0.42
During predatory bird	6 min	13.0	0.06	< 0.001	
After predatory bird	5 min	17.7	0.06	< 0.001	
**Auditory stress**					
Before thunder sound	5 min	34.5	0.04		0.01 ± 0.11
During thunder sound	1 min	22.3	0.04	< 0.001	
After thunder sound	5 min	38.9	0.03	< 0.001	

⁎ *P* <0.05 is significant difference compared to before predatory bird/thunder sound

The exposure to thunder sound induced a decrease (P < 0.001) in the hen number on the litter from 34.5 hens/4m^2^ before thunder sound exposure to 22.3 hens/4m^2^ during thunder sound exposure (Table 5). Hen numbers increased (P < 0.001) after thunder sound to 38.9 hens/4m^2^. On the long-term, hen numbers were not different (P = 0.326) after thunder sound exposure compared to before thunder sound exposure (Supplemental Table 5).

Delayed feeding decreased the hen number on the litter (P = 0.018) from 39.5 hens/4m^2^ before delayed feeding to 35.8 hens/4m^2^ during the first 5 mins of delayed feeding, and a decrease (P < 0.001) of 9.6 hens/4m^2^ during the last 5 mins compared to before delayed feeding (Table 6). After delayed feeding, hen number on the litter was lower (P < 0.001) than before delayed feeding. The same patterns were seen for the long-term response (Supplemental Table 6).

**Table 6:** **Short term and long term effect of frustrative stress (delayed feeding) on litter use of 4 experimental groups of laying hens during 10 consecutive weeks (age 38-47 weeks).** Results are given for early frustration (the first 5 mins of delayed feeding) and late frustration (the last 5 mins of delayed feeding.

**Model factors**	**Duration**	**Hens/ 4 m^2^ litter**	**SE**	P*	**Week-effect**
*Early frustration*					
Before delayed feeding	5 min	39.5	0.09		0.06 ± 0.24
During delayed feeding	First 5 min	35.8	0.04	0.018	
After delayed feeding	5 min	22.2	0.04	< 0.001	
*Late frustration*					
Before delayed feeding	5 min	39.3	0.10		0.08 ± 0.28
During delayed feeding	Last 5 min	29.7	0.04	< 0.001	
After delayed feeding	5 min	22.0	0.04	< 0.001	

⁎ *P* <0.05 is significant difference compared to before delayed feeding

As with the vertical movement analysis, the random intercepts for weeks were lower in the long-term model compared to the short-term model of litter use during visual and auditory stress, together with relatively high standard errors.

### Production

Production parameters of laying hens, averaged across the four experimental groups, are summarized for each experimental period and compared descriptively to ISA Brown layer standards (Table 7). Feed intake was consistently higher than the norm across all periods, though the magnitude of deviation fluctuated. Laying percentage remained slightly above the standard throughout the experiment. Mortality increased progressively over time relative to the norm. Body weight remained above the norm, but gradually approached it over the course of the study.

**Table 7:** **Descriptive statistics of production parameters, averaged across 4 experimental groups of laying hens per experimental period (wk 33-37; wk 38-42; wk 43-47).** Averages are compared to the norm for ISA Brown layers.

	**Pre-experimental period (wk 33 to wk 37 of age)**		**Experimental period 1 (wk 38 to wk 42 of age)**		**Experimental period 2 (wk 43 to wk 47 of age)**	
Parameter	Average	Norm deviation	Average	Norm deviation	Average	Norm deviation
**Feed intake (g /hen/d)**	131.0 ± 9.0	+ 8.0	141.0 ± 7.3	+ 7.0	131.7 ± 8.2	+ 8.7
**Egg laying percentage**	94.8 ± 6.9	+ 0.5	94.7 ± 7.0	+ 0.9	94.1 ± 8.0	+ 1.1
**Egg weight(g)**	62.9 ± 0.6	+ 0.4	63.8 ± 0.5	+ 0.7	63.5 ± 0.3	- 0.1
**Body weight (g/hen)**	1886.9 ± 33.5	+ 22.9	1914.7 ± 29.1	+ 21.8	1925.5 ± 25.4	+ 13.5
**Cumulative mortality (%)**	1.8	+ 0.3	2.9	+ 1.1	4.1	+ 1.9

## Discussion

This study investigated the potential of automated spatial behavior monitoring with cameras as a method for detecting stress events in commercial aviaries for laying hens. The findings suggest that vertical movement within the aviary and litter use are consistent spatial behavior measures across flocks over time, with quantitative differences in behavior measures between weeks that are likely caused by management interventions. Alterations in vertical movement and litter use were observed in response to acute visual and auditory stressors, as well as to delayed feeding. However, the magnitude, direction, and consistency of these alterations varied depending on the type of stressor, environmental conditions, and the time window of observation.

### Consistency of spatial behavior

A distinctive pattern was observed in flock-level vertical movement and litter use, shaped by both environmental and intrinsic factors. In the absence of management-related or experimental interventions, vertical movement remained highly consistent across weeks during afternoon resting periods. This consistency likely reflects a low-arousal state, with synchronized perching behavior as is commonly observed in group-housed hens (Appleby, 2004). The absence of strong external triggers, such as feeding, may facilitate stable resting and comfort behaviors, like dustbathing (Keeling, 1995), and thus reinforce flock-level consistency in spatial behavior. In contrast, litter use showed greater variability between weeks, even during resting periods. In this study, peaks in litter use likely reflected clustering around enrichment sites (Xu et al., 2022) (in this case, a sandbox), especially on Monday afternoons, when enrichment was replenished earlier that morning. In comparison, lower variability was seen on Friday afternoons, possibly because hens were already accustomed to the presence of new enrichment material or the enrichment was nearly depleted. These patterns suggest that litter use is more sensitive to environmental changes than vertical movement.

Flock-level litter use was more consistent during morning resting periods (9:00–10:00 h) than in the afternoon, which may reflect the diurnal behavioral patterns of laying hens. Hens typically lay eggs within the first 5 hours after lights-on, which is driven by internal biological rhythms and social facilitation (Villanueva et al., 2017). During the observed morning resting periods, most hens had likely completed egg-laying and were present on the litter to perform dustbathing or to forage. During this time, the highest maximum litter use in this study was seen, with hen occupancy reaching 10 hens/m². The consistency in litter use was disrupted by delayed feeding, which aligns with previous findings that feeding synchrony is primarily resource-driven rather than socially-mediated and that absence of feed can disrupt synchronization of feeding behavior (Collins et al., 2011). Consistency in spatial behavior might thus depend on environmental predictability.

Interestingly, exposure to auditory and visual stressors appeared to induce greater weekly consistency in vertical movement during subsequent rest periods. Although absolute movement increased slightly after stress, weekly patterns remained stable, suggesting that stress may promote synchronized vigilance or group-level arousal responses (Duranton & Gaunet, 2016). Stress may not only alter activity levels, but also reinforce coordination among hens in the flock. These findings suggest that spatial behavior under stress is dynamic, but that hens may re-establish coordinated patterns once the stressor is removed.

In summary, vertical movement appears to reflect stable flock-level behavioral rhythms, with disruptions mainly associated with feeding or experimental interventions. Interestingly, stress exposure appeared to increase consistency in vertical movement during recovery periods, possibly reflecting synchronized vigilance. In contrast, litter use is more sensitive to short-term environmental influences, suggesting it may be less reliable as a standalone indicator of flock stability. However, it could serve as a valuable metric during specific times, like in the mornings after egg-laying, when hens are more predictably engaged in activities on the litter.

### Spatial behavior measures in response to acute stress

The stress responses to the visual predatory bird, the abiotic thunder cue and the frustrative stress revealed different behavioral patterns. Exposure to a visual predator cue led to a clear suppression of vertical movement and reduced litter use, which is consistent with anti-predator behaviors, such as freezing or withdrawal to protected areas (Håkansson & Jensen, 2008). These defensive responses persisted during the immediate post-stress period (up to 5 mins). At 25 mins post-exposure, a small, but statistically significant increase in vertical movement was observed compared to pre-stress movement. While small, this may reflect exploratory activity in the aviary system, as hens assessed whether or not the threat had passed (Riber, 2012). However, litter use remained low during this time, which suggests that hens continued to avoid open spaces and did not continue performing comfort behaviors, like dustbathing (Widowski & Duncan, 2000). The strongest and most consistent behavioral response was seen during the initial predator presentation, which shows the hens sensitivity to acute threats. This is explained by evolutionary adaptations of hen for group vigilance and escape behaviors, which intensifies with flock size (Morelli et al., 2019).

In contrast to the predator cue, thunder triggered increased vertical movement and reduced litter use during exposure, indicating a generalized arousal response. In the 5 mins following thunder, both movement and litter use increased further. By 25 mins post-exposure, vertical movement decreased and litter use returned to pre-stress levels, which reflects a relatively fast behavioral recovery. These findings illustrate how loud sudden noise leads to short-term arousal without the prolonged defensive strategies seen with predator cues.

Delayed feeding caused clear changes in spatial behavior, characterized by heightened vertical movement and reduced litter use. In Compartment 1, hens responded immediately during the delay, with rapid fluctuations in litter use indicating high arousal and pacing. Pacing is often considered a frustration-related behavior which is seen, for example, when hens are deprived of nest boxes (Freire & Hughs, 1996). In Compartment 2, the response was delayed, likely due to the absence of feed-related sounds from nearby areas. This contrast shows the influence of auditory and contextual cues on behavioral expression. Feeding itself triggered a sharp spike in movement, exceeding the vertical movement performed on days without delayed feeding. Litter use remained low after feeding, which may suggest that the hens kept searching for feed in the system for a prolonged period.

Weekly variability in spatial behavior measures increased during the acute stress exposures, with especially inconsistent movement and litter use across weeks during visual and auditory stress. These fluctuations likely reflect individual differences in fear responses, coping mechanisms, and learning (Jackson et al., 2025; Manet et al., 2023), as well as sensitivity to repeated predator-like or auditory cues (Zanette et al., 2019). Taken together, spatial behavior during acute stress is stress-specific and dynamic depending on housing context, management conditions and sensitivity of the flock to stress. Future research should explore vertical movement and litter use over full 24-hour cycles to better understand how acute stressors influence spatial patterns and behavioral recovery in laying hens.

### Implementation of flock-level spatial behavior monitoring

The implementation of camera-based spatial behavior monitoring in commercial aviary systems offers promising opportunities for early detection of welfare issues at the flock level. This approach is most effective when integrating both vertical movement and litter use data, as these two spatial behavior measures together offer a more complete view of hen location within the system, their interactions with the environment, and the locations of deviations within the house (Peña Fernández et al., 2018). Monitoring strategies should be in line with the natural daily rhythms of the flock, where morning litter use patterns are ideal for detecting deviations in comfort behaviors and feeding, while afternoon periods are better for identifying stress-related changes in vertical activity (Bessei et al., 2023).

Importantly, thresholds for behavioral deviations should be established for each flock individually, as weekly variation in spatial behavior measures was lower when flocks were analyzed separately. Our results suggests that aggregating data across flocks may obscure meaningful within-flock patterns. For example, while aggregated data showed a decrease in vertical movement during thunder stress exposure, separate analyses per flock using GLMMs revealed increases in movement under thunder sound exposure. This discrepancy likely reflects inter-flock differences in baseline activity and stress responsiveness due to for example habituation to repeated stress exposure (Jackson et al., 2025). Aggregation smooths out these differences, which could mask biologically relevant responses. This supports a flock-specific, house-specific approach, where spatial behavior is compared in real-time to that flock’s historical patterns under stable management conditions. Ultimately, future research should investigate whether the magnitude of deviation from normal behavior or the speed of behavioral recovery after disruption better indicates the flock’s response to acute stress. Spatial behavior monitoring has the potential not only to improve on-farm health and welfare monitoring, but also to contribute to a deeper understanding of how laying hens navigate and adapt to their complex environments.

## CRediT authorship contribution statement

**Lara Amber van Veen:** Writing – review & editing, Writing – original draft, Visualization, Validation, Supervision, Software, Project administration, Methodology, Investigation, Formal analysis, Data curation, Conceptualization. **Anna Cornelia Maria van den Oever:** Writing – review & editing, Supervision, Resources, Methodology, Conceptualization. **Elisabeth Anna Maria Graat:** Writing – review & editing, Investigation, Formal analysis. **Tom Van Hertem:** Writing – review & editing, Supervision, Resources. **Niels Demaître:** Writing – review & editing, Supervision, Resources. **Bas Kemp:** Writing – review & editing, Visualization, Supervision, Conceptualization. **Henry van den Brand:** Writing – review & editing, Supervision, Methodology, Conceptualization.

## Disclosures

The authors declare the following financial interests/personal relationships which may be considered as potential competing interests: Lara Amber van Veen reports financial support was provided by Vencomatic Group. Anna Cornelia Maria van den Oever reports financial support was provided by Vencomatic Group. If there are other authors, they declare that they have no known competing financial interests or personal relationships that could have appeared to influence the work reported in this paper.

## Appendix Supplementary materials

Image, application 1
