# Supplementary material for: Flocks in focus: Automated video analysis of spatial behavior for stress detection in aviary-housed laying hens
Source: Poult Sci. 2025 Oct 1;104(12):105933. doi: 10.1016/j.psj.2025.105933 (PMC12547268; doi:10.1016/j.psj.2025.105933)
Supplement: Supplementary file 1 [file mmc1.docx]

**SUPPLEMENTARY MATERIAL**

**Supplemental Table 1.** **Median and IQR of vertical movements/min of 4 experimental laying hen groups during 10 consecutive weeks**. Monday: visual stressor (predator bird) was applied at 15:30 h; Wednesday: feed provision was delayed from 10:00 to 11:00 h; Friday: auditory stressor (thunder sound) was applied at 15:30 h. NA= data not available due to failed stressor or incomplete video data record.

| **Monday (predatory bird exposure)** | | | | | |
| --- | --- | --- | --- | --- | --- |
| Week | 1 | 2 | 3 | 4 | 5 |
| Group | Median (**IQR**) | | | | |
| 1 | NA | 16.0 **(8.0)** | 21.0 **(9.3)** | 16.0 **(8.3)** | NA |
| 2 | NA | 17.0 (**9.0)** | 17.0 (**10.3**) | 16.0 (**8.3**) | 17.0 (**9.0**) |
| 3 | NA | 18.0 (**8.0**) | 20.5 (**10.3**) | 13.0 (**9.0**) | 14.0 (**8.0**) |
| 4 | NA | 15.0 (**9.0**) | 20.0 (**9.0**) | 16.0 (**10.0**) | 15.5 (**10.0**) |
| Week | 6 | 7 | 8 | 9 | 10 |
| Group | Median (**IQR**) | |  |  |  |
| 1 | 14.0 (**7.3)** | 15.0 (**9.3**) | NA | 14.5 (**11.0)** | 13.0 (**8.0**) |
| 2 | 14.0 (**9.0**) | 16.0 (**10.3**) | 16.0 (**9.0**) | 14.0 (**10.0**) | 13.0 (**8.2**) |
| 3 | 12.5 (**9.3**) | 15.0 (**9.3**) | 13.0 (**10.0**) | 14.5 (**11.3**) | 14.0 (**9.0**) |
| 4 | 14.0 (**7.0**) | NA | 14.0 (**10.0**) | 16.5 (**9.3**) | 15.5 (**9.0**) |
| **Wednesday (delayed feeding)** | | | | |  |
| Week | 1 | 2 | 3 | 4 | 5 |
| Group | Median (**IQR**) | |  |  |  |
| 1 | 11.0 (**8.0**) | NA | 10.0 (**6.3**) | 11.0 (**10.0**) | 10.0 (**6.3**) |
| 2 | 10.0 (**8.0**) | NA | 10.0 (**7.0**) | 10.5 (**8.0**) | 10.0 (**6.0**) |
| 3 | 9.0 (**7.0**) | NA | 10.0 (**6.3**) | 9.0 (**7.3**) | 10.0 (**7.3**) |
| 4 | 9.0 (**6.0**) | NA | 10.0 (**6.0**) | 9.5 (**8.0**) | 9.0 (**5.0**) |
| Week | 6 | 7 | 8 | 9 | 10 |
| Group | Median (**IQR**) | |  |  |  |
| 1 | 11.0 (**7.0**) | NA | 10.0 (**6.3**) | 9.0 (**6.0**) | 9.0 (**6.0**) |
| 2 | NA | 10.0 (**6.0**) | 10.0 (**7.0**) | 10.0 (**6.3**) | 9.0 (**6.0**) |
| 3 | NA | NA | 10.0 (**6.0**) | 8.0 (**4.0**) | 10.0 (**6.3**) |
| 4 | NA | NA | 10.0 (**5.0**) | 10.0 (**6.0**) | 10.0 (**6.0**) |
| **Friday (thunder sound exposure)** | | | | | |
| Week | 1 | 2 | 3 | 4 | 5 |
| Group | Median (**IQR**) | |  |  |  |
| 1 | 16.0 (**8.3**) | 15.0 (**9.3**) | 15.5 (**11.3**) | 16 (**10.5**) | 16 (**7.3**) |
| 2 | 14.0 (**8.0**) | 14.0 (**6.3**) | 14.0 (**7.3**) | 14.0 (**9.0**) | 15.0 (**8.0**) |
| 3 | 16.0 (**7.3**) | 16.0 (**8.0**) | 15.0 (**8.0**) | 14.0 (**8.0**) | 15.0 (**10.3**) |
| 4 | 16.0 (**11.0**) | 16.0 (**7.0**) | 15.0 (**9.3**) | 15.5 (**9.0**) | 16.0 (**7.3**) |
| Week | 6 | 7 | 8 | 9 | 10 |
| Group | Median (**IQR**) | |  |  |  |
| 1 | 17.0 (**10.0**) | 14.0 (**9.3**) | 15.0 (**9.0**) | NA | 16.0 (**8.0**) |
| 2 | 14.0 (**7.3**) | 14.5 (**7.0**) | 13.0 (**6.0**) | NA | 11.0 (**7.0**) |
| 3 | 14.5 (**8.0**) | 15.0 (**7.0**) | 16.0 (**7.0**) | 15.0 (**8.0**) | 14.0 (**8.3**) |
| 4 | 14.0 (**7.0**) | 15.0 (**9.0**) | 15.0 (**10.0**) | 16.5 (**7.0**) | 14.6 (**7.0**) |

**Supplemental Table 2.** **Median and IQR of hen numbers/4 m^2^ litter of 4 experimental laying hen groups during 10 consecutive weeks.** Monday: visual stressor was applied at 15:30 h; Wednesday: feed provision was delayed from 10:00 to 11:00 h; Friday: auditory stressor was applied at 15:30 h. NA= data not available due to failed stressor or incomplete video data record.

| **Monday (predatory bird exposure)** | | | | | |
| --- | --- | --- | --- | --- | --- |
| Week | 1 | 2 | 3 | 4 | 5 |
| Group | Median (**IQR**) | |  |  |  |
| 1 | NA | 38.0 (**9.0**) | 40.0 (**11.0**) | 27.0 (**10.0**) | NA |
| 2 | NA | 51.0 (**10.0**) | 35.0 (**14.0**) | 28.0 (**10.0**) | 25.0 (**9.0**) |
| 3 | NA | 43.0 (**10.0**) | 44.0 (**9.0**) | 25.0 (**11.0**) | 22.0 (**12.0**) |
| 4 | NA | 50.0 (**10.0**) | 48.0 (**10.0**) | 34.0 (**9.0**) | 28.0 (**10.0**) |
| Week | 6 | 7 | 8 | 9 | 10 |
| Group | Median (**IQR**) | |  |  |  |
| 1 | 23.0 (**9.0**) | 19.0 (**11.0**) | NA | 17.0 (**7.0**) | 46.0 (**10.0**) |
| 2 | 25.0 (**10.0**) | 21.0 (**9.0**) | 19.0 (**11.0**) | 25.0 (**10.0**) | 44.0 **(11.0)** |
| 3 | 22.0 (**10.0**) | 24.0 (**9.0**) | 25.0 (**11.0**) | 26.0 (**11.0**) | 47.0 (**16.0**) |
| 4 | 31.0 (**11.0**) | NA | 22.0 (**8.0**) | 34.0 (**10.0**) | 50.0 (**11.0**) |
| **Wednesday (delayed feeding)** | | |  |  |  |
| Week | 1 | 2 | 3 | 4 | 5 |
| Group | Median (**IQR**) | |  |  |  |
| 1 | 64.0 (**27.0**) | NA | 35.0 (**20.0**) | 29.0 (**28.0**) | 20.0 (**16.0**) |
| 2 | 72.0 (**15.0**) | NA | 49.0 (**17.0**) | 38.0 (**31.0**) | 33.0 (**18.0**) |
| 3 | 48.0 (**28.0**) | NA | 29.0 (**14.0**) | 32.0 (**25.0**) | 24.0 (**20.0**) |
| 4 | 65.0 (**12.0**) | NA | 39.0 (**10.0**) | 41.0 (**25.0**) | 34.0 (**21.0**) |
| Week | 6 | 7 | 8 | 9 | 10 |
| Group | Median (**IQR**) | |  |  |  |
| 1 | NA | NA | NA | 17.0 (**11.0**) | 30.0 (**14.0**) |
| 2 | NA | 36.0 (**15.0**) | 30.0 (**21.0**) | 36.0 (**15.0**) | 46.0 (**14.0**) |
| 3 | 22.0 (**18.0**) | NA | 25.0 (**17.0**) | 28.0 (**15.0**) | 31.0 (**17.0**) |
| 4 | 30.0 (**21.0**) | NA | 36.0 (**19.0**) | 38.0 (**14.0**) | 45.0 (**12.0**) |
| **Friday (thunder sound exposure)** | | | | | |
| Week | 1 | 2 | 3 | 4 | 5 |
| Group | Median (**IQR**) | |  |  |  |
| 1 | 31.0 (**10.0**) | 30.0 (**11.0**) | 32.0 (**17.0**) | 25.0 (**10.0**) | 23.0 (**12.0**) |
| 2 | 31.0 (**12.0**) | 32.0 (**10.0**) | 35.0 (**11.0**) | 23.0 (**8.0**) | 22.0 (**10.0**) |
| 3 | 33.0 (**9.0**) | 29.0 (**10.0**) | 33.0 (**9.0**) | 29.0 (**9.0**) | 26.0 (**10.0**) |
| 4 | 43.0 (**11.0**) | 38.0 **(9.0)** | 36.0 (**12.0**) | 34.0 (**8.0**) | 33.0 (**11.0**) |
| Week | 6 | 7 | 8 | 9 | 10 |
| Group | Median (**IQR**) | |  |  |  |
| 1 | 20.0 (**11.0**) | 26.0 (**14.0**) | 21.0 (**9.0**) | NA | 33.0 (**13.0**) |
| 2 | 23.0 (**8.0**) | 24.0 (**8.0**) | 24.0 (**7.0**) | NA | 27.0 (**9.0**) |
| 3 | 26.5 (**8.0**) | 33.0 (**10.0**) | 34.0 (**8.0**) | 30.0 (**10.0**) | 38.0 (**9.0**) |
| 4 | 31.0 (**9.0**) | 30.0 (**10.0**) | 39.0 (**11.0**) | 34.0 (**10.0**) | 43.0 (**11.0**) |

**Supplemental Table 3.** **Long term effect of visual (predator bird) and auditory (thunder sound) stressors on vertical movement of 4 experimental groups of laying hens during 10 consecutive weeks (age 38-47 weeks).**

| **Stressor** | **Duration** | **Movement/ min** | **SE** | ***P**** | **Week-effect** |
| --- | --- | --- | --- | --- | --- |
| **Visual stress** |  |  |  |  |  |
| Before predatory bird | 25 min | 13.6 | 0.04 |  | 0.02 ± 0.13 |
| During predatory bird | 6 min | 7.7 | 0.04 | < 0.001 |  |
| After predatory bird | 25 min | 14.5 | 0.02 | 0.004 |  |
| **Auditory stress** |  |  |  |  |  |
| Before thunder sound | 25 min | 7.3 | 0.05 |  | 0.00 ± 0.02 |
| During thunder sound | 1 min | 15.1 | 0.05 | < 0.001 |  |
| After thunder sound | 25 min | 12.8 | 0.052 | < 0.001 |  |
| * P<0.05 is significant difference compared to before predatory bird/thunder sound | | | | | |

**Supplemental Table 4**. **Long term effect of frustrative stress (delayed feeding) on vertical movement of 4 experimental groups of laying hens during 10 consecutive weeks (age 38-47 weeks).** Results are given for early frustration (the first 30 mins of delayed feeding) and later frustration (the last 30 mins of delayed feeding.

| **Model factors** | **Duration** | **Movement/ min** | **SE** | ***P**** | **Week-effect** | |
| --- | --- | --- | --- | --- | --- | --- |
| *Early frustration* |  |  |  |  |  | |
| Before delayed feeding | 30 min | 8.3 | 0.03 |  | 0.004 +/- 0.06 | |
| During delayed feeding | First 30 min | 9.6 | 0.02 | < 0.001 |  |  |
| After delayed feeding | 30 min | 10.3 | 0.02 | < 0.001 |  |  |
| *Late frustration* |  |  |  |  |  | |
| Before delayed feeding | 30 min | 8.3 | 0.02 |  | 0.001 +/- 0.04 | |
| During delayed feeding | Last 30 min | 9.7 | 0.02 | < 0.001 |  | |
| After delayed feeding | 30 min | 10.4 | 0.02 | < 0.001 |  | |
| * P<0.05 is significant difference compared to before delayed feeding | | | | | |  |

**Supplemental Table 5.** **Long term effect of visual (predator bird) and auditory (thunder sound) stressors on litter use of 4 experimental groups of laying hens during 10 consecutive weeks (age 38-47 weeks).**

| **Model factors** | **Duration** | **Hens/4 m^2^ litter** | **SE** | ***P**** | **Week-effect** |  |
| --- | --- | --- | --- | --- | --- | --- |
| **Visual stress** |  |  |  |  |  |  |
| Before predatory bird | 25 min | 30.8 | 0.10 |  | 0.10 ± 0.31 |  |
| During predatory bird | 6 min | 13.4 | 0.03 | < 0.001 |  |  |
| After predatory bird | 25 min | 28.0 | 0.02 | < 0.001 |  |  |
| **Auditory stress** |  |  |  |  |  |  |
| Before thunder sound | 25 min | 31.9 | 0.03 |  | 0.01 ± 0.10 |  |
| During thunder sound | 1 min | 22.8 | 0.03 | < 0.001 |  |  |
| After thunder sound | 25 min | 32.2 | 0.01 | 0.326 |  |  |
| * P<0.05 is significant difference compared to before predatory bird/thunder sound | | | | | | |

**Supplemental Table 6.** **Long term effect of frustrative stress (delayed feeding) on litter use of 4 experimental groups of laying hens during 10 consecutive weeks (age 38-47 weeks).** Results are given for early frustration (the first 30 mins of delayed feeding) and later frustration (the last 30 mins of delayed feeding.

| **Model factors** | **Duration** | **Hens/ 4 m^2^ litter** | **SE** | ***P**** | **Week-effect** | |  |
| --- | --- | --- | --- | --- | --- | --- | --- |
| *Early frustration* |  |  |  |  |  | |  |
| Before delayed feeding | 30 min | 39.7 | 0.09 |  | 0.08 ± 0.28 | |  |
| During delayed feeding | First 30 min | 32.8 | 0.02 | < 0.001 |  |  |  |
| After delayed feeding | 30 min | 18.2 | 0.02 | < 0.001 |  |  |  |
| *Later frustration* |  |  |  |  |  | |  |
| Before delayed feeding | 30 min | 40.1 | 0.11 |  | 0.10 ± 0.31 | |  |
| During delayed feeding | Last 30 min | 28.8 | 0.02 | < 0.001 |  |  |  |
| After delayed feeding | 30 min | 18.2 | 0.02 | < 0.001 |  |  |  |
| * P<0.05 is significant difference compared to before delayed feeding | | | | | | | |
